# Supplementary material for: Galectins as Checkpoints of the Immune System in Cancers, Their Clinical Relevance, and Implication in Clinical Trials
Source: Biomolecules. 2020 May 12;10(5):750. doi: 10.3390/biom10050750 (PMC7277089; doi:10.3390/biom10050750)
Supplement: Supplementary file 1 [file biomolecules-10-00750-s001.pdf]

biomolecules

MDPI

Table S1. Clinical trials implicating galectins as biomarkers (1).

CLINICAL TRIAL NUMBERS	TITLE	STATUS	TARGETS	BIOMARKERS	TUMOR TYPES	PHASES	RESULTS	START DATE	END DATE
NCT02530125	Pidilizumab in Treating Patients With Stage III-IV Diffuse Large B-Cell Lymphoma Following First Remission	Terminated	PD-1	Gal-1 Biomarker	Lymphoma	Phase 2	Stopped without public explanation.	Sep 2015	Jan 2017
NCT02605512	Breast Cancer and Cardiotoxicity Induced by RadioTherapy: the BACCARAT Study	Recruiting	Radiotherapy	Gal-3 Biomarker	Breast Cancer	Not Applicable		Oct 2015	Sep 2020
NCT02494453	Pilot Study of Biomarkers and Cardiac MRI as Early Indicators of Cardiac Exposure Following Breast Radiotherapy	Terminated	Radiotherapy	biomarker	Breast Cancer			June 2015	July 2017
NCT02496260	Biomarkers and Cardiac MRI as Early Indicators of Cardiac Exposure Following Breast Radiotherapy	Unknown status	Radiotherapy	Gal-3 Biomarker	Breast Cancer			July 2015	
NCT03297346	Early Detection of Cardiovascular Changes After Radiotherapy for Breast Cancer	Recruiting	Radiotherapy	Gal-3 Biomarker	Breast Cancer Female	Not Applicable		Sep 2017	May 2021
NCT01347970	Pharmacologic Reversal of Ventricular Remodeling in Childhood Cancer Survivors at Risk for Congestive Heart Failure (PREVENT-CHF): A Phase IIB Randomized Placebo-Controlled Trial	Active, not recruiting	multiple	Gal-3 Biomarker	Cancer Survivor	Phase 2		May 2012	Dec 2020
NCT01511653	Validation of Colon Biomarkers for the Early Detection of Colorectal Adenocarcinoma	Completed	-	Gal-3 Biomarker	Colon Cancer|Rectal Cancer			Oct 2011	April 2019
NCT00388700	A New Agent GM-CT-01 in Combination With 5-FU, Avastin and Leucovorin in Subjects With Colorectal Cancer	Withdrawn	Galectins	Gal-3 Biomarker	Colorectal Cancer	Phase 2		Oct 2006	
NCT02717507	Carvedilol in Preventing Heart Failure in Childhood Cancer Survivors	Recruiting	Beta-blockers	Gal-3 Biomarker	Malignant Neoplasm	Phase 2		April 2016	

Table S2. Clinical trials implicating galectins as biomarkers (2).

CLINICAL TRIAL NUMBERS	TITLE	STATUS	TARGETS	BIOMARKERS	TUMOR TYPES	PHASES	RESULTS	START DATE	END DATE
NCT02117362	Galectin Inhibitor (GR-MD-02) and Ipilimumab in Patients With Metastatic Melanoma	Completed	PD-1	Gal-3 Biomarker	Metastatic Melanoma	Phase 1		May 2014	Nov 2018
NCT03488134	Predicting Prognosis and Recurrence of Thyroid Cancer Via New Biomarkers, Urinary Exosomal Thyroglobulin and Galectin-3	Active, not recruiting	Gal-3 Pronostic	Gal-3 Biomarker	Thyroid Cancer			Aug 3, 2018	July 31, 2023
NCT01896999	Brentuximab Vedotin and Nivolumab With or Without Ipilimumab in Treating Patients With Relapsed or Refractory Hodgkin Lymphoma	Recruiting	PD-1 +CTLA-4 +CD30	Biomarkers (Gal-1+Tim-3)	Lymphoma	Phase 1|Phase 2		Jan 24, 2014	
NCT03940352	HDM201 in Combination With MBG453 or Venetoclax in Patients With Acute Myeloid Leukemia (AML) or High-risk Myelodysplastic Syndrome (MDS)	Recruiting	P53	Tim-3 Biomarker	AML	Phase 1		June 24, 2019	Apr 22, 2021
NCT03289819	Neoadjuvant Pembrolizumab(Pbr)/Nab-Paclitaxel Followed by Pbr/Epirubicin/Cyclophosphamide in TNBC	Active, not recruiting	PD-1 + Chemoth	Tim-3 Biomarker	Breast Cancer	Phase 2		Mar 23, 2018	Sep 2020
NCT02303366	Pilot Study of Stereotactic Ablation for Oligometastatic Breast Neoplasia in Combination With the Anti-PD-1 Antibody MK-3475	Completed	PD-1	Tim-3 Biomarker	Breast Neoplasms|Bone Neoplasms	Phase 1		Sep 2015	Apr 2017
NCT03661047	OMega-3 Fatty Acid for the Immune Modulation of Colon Cancer	Recruiting	Metabolism	Tim-3 Biomarker	Colon Cancer	Phase 2		Nov 30, 2019	Sep 2023
NCT04001101	Anti-PD-1 +/- RT for MSI-H Solid Tumors	Recruiting	PD-1	Tim-3 Biomarker	Colorectal Cancer	Phase 2		Oct 10, 2019	July 2021
NCT03609424	PDR001 Plus Imatinib for Metastatic or Unresectable GIST	Recruiting	PD-1	Tim-3 Biomarker	Gastrointestinal Stromal Tumors	Phase 1|Phase 2		Feb 14, 2019	Aug 30, 2020

Table S3. Clinical trials implicating galectins as biomarkers (3).

CLINICAL TRIAL NUMBERS	TITLE	STATUS	TARGETS	BIOMARKERS	TUMOR TYPES	PHASES	RESULTS	START DATE	END DATE
NCT03961971	Trial of Anti-Tim-3 in Combination With Anti-PD-1 and SRS in Recurrent GBM	Recruiting	PD-1	Tim-3 Biomarker	Glioblastoma Multiforme	Phase 1		Feb 18, 2020	Feb 2024
NCT03066648	Study of PDR001 and/or MBG453 in Combination With Decitabine in Patients With AML or High Risk MDS	Recruiting	PD-1	Tim-3 Biomarker	Leukemia	Phase 1		Oct 29, 2010	Apr 1, 2021
NCT03680508	TSR-022 (Anti-TIM-3 Antibody) and TSR-042 (Anti-PD-1 Antibody) in Patients With Liver Cancer	Recruiting	-	Tim-3 Biomarker	Liver Cancer	Phase 2		Nov 4, 2019	Oct 2023
NCT03549546	Expression of the Inhibitory Receptors on Lymphocytes T Cells After Lung Cancer Surgery.	Completed	Surgery	Tim-3 Biomarker	Lung Cancer	Not Applicable		June 14, 2018	Feb 2019
NCT02846103	Study of Anti-telomerase T CD4 Immunity in Metastatic Lung Cancer	Unknown status	-	Tim-3 Biomarker	Lung Cancer	Not Applicable		Dec 2015	Dec 2019
NCT03489343	Sym023 (Anti-TIM-3) in Patients With Advanced Solid Tumor Malignancies or Lymphomas	Active, not recruiting	-	Tim-3 Biomarker	Lymphoma	Phase 1		May 30, 2018	Sep 2020
NCT03311412	Sym021 Monotherapy and in Combination With Sym022 or Sym023 in Patients With Advanced Solid Tumor Malignancies or Lymphomas	Recruiting	PD-1	Tim-3 Biomarker	Lymphoma	Phase 1		Dec 7, 2017	Nov 2020
NCT03873805	PSCA-CAR T Cells in Treating Patients With PSCA+ Metastatic Castration Resistant Prostate Cancer	Recruiting	CART	Tim-3 Biomarker	mCRPC	Phase 1		May 23, 2019	Feb 2021
NCT03772899	Fecal Microbial Transplantation in Combination With Immunotherapy in Melanoma Patients (MIMic)	Recruiting	Fecal + Immunoth	Tim-3 Biomarker	Melanoma	Phase 1		March 27, 2019	Dec 2023
NCT03089606	Pembrolizumab TX-naive Distant Mets Melanoma and Use of AMT (PET) at Baseline as Imaging Biomarker	Recruiting	PD-1	Tim-3 Biomarker	Melanoma	Phase 2		June 1, 2017	June 2022

Table S4. Clinical trials implicating galectins as biomarkers (4).

CLINICAL TRIAL NUMBERS	TITLE	STATUS	TARGETS	BIOMARKERS	TUMOR TYPES	PHASES	RESULTS	START DATE	END DATE
NCT03961971	Trial of Anti-Tim-3 in Combination With Anti-PD-1 and SRS in Recurrent GBM	Recruiting	PD-1	Tim-3 Biomarker	Glioblastoma Multiforme	Phase 1		Febr 18, 2020	Feb 2024
NCT03066648	Study of PDR001 and/or MBG453 in Combination With Decitabine in Patients With AML or High Risk MDS	Recruiting	PD-1	Tim-3 Biomarker	Leukemia	Phase 1		Oct 29, 2010	Apr 1, 2021
NCT03680508	TSR-022 (Anti-TIM-3 Antibody) and TSR-042 (Anti-PD-1 Antibody) in Patients With Liver Cancer	Recruiting	-	Tim-3 Biomarker	Liver Cancer	Phase 2		Nov 4, 2019	Oct 2023
NCT03549546	Expression of the Inhibitory Receptors on Lymphocytes T Cells After Lung Cancer Surgery.	Completed	Surgery	Tim-3 Biomarker	Lung Cancer	Not Applicable		June 14, 2018	Feb 2019
NCT02846103	Study of Anti-telomerase T CD4 Immunity in Metastatic Lung Cancer	Unknown status	-	Tim-3 Biomarker	Lung Cancer	Not Applicable		Dec 2015	Dec 2019
NCT03489343	Sym023 (Anti-TIM-3) in Patients With Advanced Solid Tumor Malignancies or Lymphomas	Active, not recruiting	-	Tim-3 Biomarker	Lymphoma	Phase 1		May 30, 2018	Sep 2020
NCT03311412	Sym021 Monotherapy and in Combination With Sym022 or Sym023 in Patients With Advanced Solid Tumor Malignancies or Lymphomas	Recruiting	PD-1	Tim-3 Biomarker	Lymphoma	Phase 1		Dec 7, 2017	Nov 2020
NCT03873805	PSCA-CAR T Cells in Treating Patients With PSCA+ Metastatic Castration Resistant Prostate Cancer	Recruiting	CART	Tim-3 Biomarker	mCRPC	Phase 1		May 23, 2019	Feb 2021
NCT03772899	Fecal Microbial Transplantation in Combination With Immunotherapy in Melanoma Patients (MIMic)	Recruiting	Fecal Th+ Immunoth	Tim-3 Biomarker	Melanoma	Phase 1		Mar 27, 2019	Dec 2023
NCT03089606	Pembrolizumab TX-naive Distant Mets Melanoma and Use of AMT (PET) at Baseline as Imaging Biomarker	Recruiting	PD-1	Tim-3 Biomarker	Melanoma	Phase 2		June 1, 2017	June 2022

Table S5. Clinical trials implicating galectins as biomarkers (5).

CLINICAL TRIAL NUMBERS	TITLE	STATUS	TARGETS	BIOMARKERS	TUMOR TYPES	PHASES	RESULTS	START DATE	END DATE
NCT04253145	Study to Assess Safety,Tolerability,Efficacy of PM01183 and Atezolizumab in Patients w/ Advanced Small Cell Lung Cancer.	Recruiting	PD-L1 + DNA	Tim-3 Biomarker	NSCLC	Phase 1		Dec 13, 2019	Feb 2022
NCT03121417	Nivolumab in Treating Patients With Advanced Metastatic Non-small Cell Lung Cancer	Withdrawn	PD-1	Tim-3 Biomarker	NSCLC	Phase 2		July 21, 2017	Mar 2019
NCT01567891	CT Antigen TCR-redirected T Cells for Ovarian Cancer.	Completed	Immunoth	Tim-3 Biomarker	Ovarian Cancer	Phase 1| Phase 2		July 9, 2013	June 2017
NCT03618355	Trial of eRapa in Prostate Cancer Patients	Active, not recruiting		Tim-3 Biomarker	Pca	Phase 1		July 20, 2018	July 2020
NCT02636725	Axitinib and Pembrolizumab in Subjects With Advanced Alveolar Soft Part Sarcoma and Other Soft Tissue Sarcomas	Active, not recruiting	PD-1 +Chemoth	Tim-3 Biomarker	Sarcomas	Phase 2		April 19, 2016	March 2021
NCT03946943	Study of Anlotinib Hydrochloride and Toripalimab in Subjects With Unresectable or Metastatic Undifferentiated Pleomorphic Sarcoma	Not yet recruiting	PD-1	Tim-3 Biomarker	Sarcomas	Phase 2		July 19, 2019	July 2023
NCT03099109	A Study of LY3321367 Alone or With LY3300054 in Participants With Advanced Relapsed/Refractory Solid Tumors	Active, not recruiting	PD-L1	Tim-3 Biomarker	Solid Tumor	Phase 1		April 12, 2017	June 2020
NCT03752177	A Study of LY3415244 in Participants With Advanced Solid Tumors	Terminated	PD-L1	Tim-3 Biomarker	Solid Tumor	Phase 1	Stopped without public explanation.	Nov 22, 2018	Oct 2019
NCT03744468	Study of BGB-A425 in Combination With Tislelizumab in Advanced Solid Tumors	Recruiting	-	Tim-3 Biomarker	Solid tumors	Phase 1| Phase 2		Nov 13, 2018	Dec 2021

Table S6. Clinical trials implicating galectins as biomarkers (6).

CLINICAL TRIAL NUMBERS	TITLE	STATUS	TARGETS	BIOMARKERS	TUMOR TYPES	PHASES	RESULTS	START DATE	END DATE
NCT01100944	A Phase 1/2 Study of PXD101 (Belinostat) in Combination With Cisplatin, Doxorubicin and Cyclophosphamide in the First Line Treatment of Advanced or Recurrent Thymic, Malignancies	Terminated	HDAC +Radioth +Chemoth	Tim-3 Biomarker	Thymic malignacies	Phase 1| Phase 2	2 out of 6 patients experienced a dose limiting toxicity (DLT).	Mar 4, 2010	Oct 2015
NCT03307785	Study of Niraparib, TSR-022, Bevacizumab, and Platinum-Based Doublet Chemotherapy in Combination With TSR-042	Active, not recruiting	PD-1 + VEGF +Chemoth	Tim-3 Biomarker	Various	Phase 1		Oct 12, 2017	Feb 2021
NCT03058289	A Phase 1/2 Safety Study of Intratumorally Dosed INT230-6	Recruiting	Chemoth	Tim-3 Biomarker	Various	Phase 1| Phase 2		Feb 9, 2017	Oct 2022
NCT03595124	A Study to Compare Treatments for a Type of Kidney Cancer Called TFE/Translocation Renal Cell Carcinoma (tRCC)	Recruiting	multiple	Tim-3 Biomarker	Various	Phase 2		Oct 9, 2018	Jan 9, 2024
NCT03684785	Intratumoral AST-008 Combined With Pembrolizumab in Patients With Advanced Solid Tumors	Recruiting	PD-1 + Anti-TLR9	Tim-3 Biomarker	Various	Phase 1| Phase 2		Dec 13, 2018	Apr 2021

Table S7. Clinical trials using galectins inhibitors (1).

CLINICAL TRIAL NUMBERS	TITLE	STATUS	TARGETS	BIOMARKERS	TUMOR TYPES	PHASES	RESULTS	START DATE	END DATE
NCT01724320	A Phase 1, First-in-man Study of OTX008 Given Subcutaneously as a Single Agent to Patients With Advanced Solid Tumors	Unknown status	Gal-1		Solid Tumors	Phase 1		Feb 2012	May 2013
NCT00388700	A New Agent GM-CT-01 in Combination With 5-FU, Avastin and Leucovorin in Subjects With Colorectal Cancer	Withdrawn	Galectins	Gal-3 Biomarker	Colorectal Cancer	Phase 2		Oct 2006	
NCT00776802	GCS-100LE in Combination With Etoposide and Dexamethasone in Relapsed or Refractory Diffuse Large B-Cell Lymphoma	Withdrawn	Gal-3		Lymphoma	Phase 1 Phase 2		July 2008	Apr 2009
NCT01723813	Peptide Vaccinations Plus GM-CT-01 in Melanoma	Terminated	Gal-3 +Vaccine		Metastatic Melanoma	Phase 1 Phase 2	[1–2]	Apr 2012	Apr 2015
NCT01681823	Effect of Modified Citrus Pectin on PSA Kinetics in Biochemical Relapsed PC With Serial Increases in PSA	Completed	Gal-3		Prostatic Neoplasms	Phase 2	[3–8]	June 2013	Jan 2020

Table S8. Clinical trials using galectins inhibitors (2).

CLINICAL TRIAL NUMBERS	TITLE	STATUS	TARGETS	BIOMARKERS	TUMOR TYPES	PHASES	RESULTS	START DATE	END DATE
NCT01100944	A Phase 1/2 Study of PXD101 (Belinostat) in Combination With Cisplatin, Doxorubicin and Cyclophosphamide in the First Line Treatment of Advanced or Recurrent Thymic, Malignancies	Terminated	HDAC +Radioth +Chemoth	Tim-3 Biomarker	Thymic malignacies	Phase 1 Phase 2	2 out of 6 patients experienced a dose limiting toxicity (DLT).	Mar 4, 2010	Oct 2015
NCT03307785	Study of Niraparib, TSR-022, Bevacizumab, and Platinum-Based Doublet Chemotherapy in Combination With TSR-042	Active, not recruiting	PD-1 + VEGF +Chemoth	Tim-3 Biomarker	Various	Phase 1		Oct 12, 2017	Febr 2021
NCT03058289	A Phase 1/2 Safety Study of Intratumorally Dosed INT230-6	Recruiting	Chemoth	Tim-3 Biomarker	Various	Phase 1 Phase 2		Feb 9, 2017	Oct 2022
NCT03595124	A Study to Compare Treatments for a Type of Kidney Cancer Called TFE/Translocation Renal Cell Carcinoma (tRCC)	Recruiting	multiple	Tim-3 Biomarker	Various	Phase 2		Oct 9, 2018	Jan 2024
NCT03684785	Intratumoral AST-008 Combined With Pembrolizumab in Patients With Advanced Solid Tumors	Recruiting	PD-1 + Anti-TLR9	Tim-3 Biomarker	Various	Phase 1 Phase 2		Dec 13, 2018	Apr 2021

Table S9. Clinical trials using galectins inhibitors (3).

CLINICAL TRIAL NUMBERS	TITLE	STATUS	TARGETS	BIOMARKERS	TUMOR TYPES	PHASES	RESULTS	START DATE	END DATE
NCT02817633	A Phase 1 Study of TSR-022, an Anti-TIM-3 Monoclonal Antibody, in Patients With Advanced Solid Tumors (AMBER)	Recruiting	TIM-3		Solid Tumors	Phase 1		July 2016	June 2022
NCT03652077	A Safety and Tolerability Study of INCAGN02390 in Select Advanced Malignancies	Recruiting	TIM-3		Various	Phase 1		Aug 29, 2018	Jan 2021
NCT00110721	GM-CT-01 Plus 5-Fluorouracil as Third- or Fourth-Line Therapy for Metastatic Colorectal Cancer	Terminated	Galectins + ADN		Colorectal Cancer	Phase 2	Stopped without public explanation.	May 2005	Mar 2012
NCT00054977	Safety of GM-CT-01 With and Without 5-Fluorouracil in Patients With Solid Tumors	Completed	Galectins + DNA		various	Phase 1		Feb 2003	
NCT02117362	Galectin Inhibitor (GR-MD-02) and Ipilimumab in Patients With Metastatic Melanoma	Completed	Galectins (Gal-3) + PD-1		Metastatic Melanoma	Phase 1	Stopped and Continued by NCT02575404	May 8, 2014	Nov 2018
NCT02575404	GR-MD-02 Plus Pembrolizumab in Melanoma, Non-small Cell Lung Cancer, and Squamous Cell Head and Neck Cancer Patients	Recruiting	Galectins (Gal-3) + PD-1		Various	Phase 1		May 16, 2016	

Reference

1. Demotte, N.; Bigirimana, R.; Wieers, G.; Stroobant, V.; Squifflet, J. L.; Carrasco, J.; Thielemans, K.; Baurain, J. F.; Van Der Smissen, P.; Courtoy, P. J.; et al. A short treatment with galactomannan GM-CT-01 corrects the functions of freshly isolated human tumor-infiltrating lymphocytes. *Clin. Cancer Res.* **2014,** *20*, 1823–1833.
2. Demotte, N.; Wieers, G.; Van Der Smissen, P.; Moser, M.; Schmidt, C.; Thielemans, K.; Squifflet, J. L.; Weynand, B.; Carrasco, J.; Lurquin, C.; et al. A Galectin-3 Ligand Corrects the Impaired Function of Human CD4 and CD8 Tumor-Infiltrating Lymphocytes and Favors Tumor Rejection in Mice. *Cancer Res.* **2010,** *70*, 7476–7488.
3. Abu-Elsaad, N. M.; Elkashef, W. F. Modified citrus pectin stops progression of liver fibrosis by inhibiting galectin-3 and inducing apoptosis of stellate cells. *Can. J. Physiol. Pharmacol.* **2016,** *94*, 554-562.
4. Delphi, L.; Sepehri, H.; Khorramizadeh, M. R.; Mansoori, F. Pectic-Oligoshaccharides from Apples Induce Apoptosis and Cell Cycle Arrest in MDA-MB-231 Cells, a Model of Human Breast Cancer. *Asian Pac. J. Cancer Prev.* **2015,** *16*, 5265–5271.
5. Ahmad, N.; Gabius, H. J.; Andre, S.; Kaltner, H.; Sabesan, S.; Roy, R.; Liu, B.; Macaluso, F.; Brewer, C. F. Galectin-3 precipitates as a pentamer with synthetic multivalent carbohydrates and forms heterogeneous cross-linked complexes. *J. Biol. Chem.* **2004,** *279*, 10841-10847.
6. Chauhan, D.; Li, G.; Podar, K.; Hideshima, T.; Neri, P.; He, D.; Mitsiades, N.; Richardson, P.; Chang, Y.; Schindler, J.; et al. A novel carbohydrate-based therapeutic GCS-100 overcomes bortezomib resistance and enhances dexamethasone-induced apoptosis in multiple myeloma cells. *Cancer Res.* **2005,** *65*, 8350-8358.
7. Traber, P. G.; Chou, H.; Zomer, E.; Hong, F.; Klyosov, A.; Fiel, M. I.; Friedman, S. L. Regression of fibrosis and reversal of cirrhosis in rats by galectin inhibitors in thioacetamide-induced liver disease. *PLoS ONE* **2013,** *8*, e75361.
8. Traber, P. G.; Zomer, E. Therapy of experimental NASH and fibrosis with galectin inhibitors. *PLoS ONE* **2013,** *8*, e83481.
